# Structurally nanoengineered antimicrobial peptide polymers: design, synthesis and biomedical applications

**DOI:** 10.1007/s11274-021-03109-z

**Published:** 2021-07-19

**Authors:** Ronisha Ramamurthy, Chetan H. Mehta, Usha Y. Nayak

**Affiliations:** Department of Pharmaceutics, Manipal College of Pharmaceutical Sciences, Manipal Academy of Higher Education, 576104 Manipal, Karnataka India

**Keywords:** Antimicrobial resistance, Bio-imaging, Drug delivery, Reversible addition fragmentation chain transfer (RAFT), Ring-opening polymerization (ROP), Structurally nano engineered antimicrobial peptide polymers (SNAPPs)

## Abstract

**Abstract:**

Antimicrobial resistance not only increases the contagiousness of infectious diseases but also a threat for the future as it is one of the health care concern around the globe. Conventional antibiotics are unsuccessful in combating chronic infections caused by multidrug-resistant (MDR) bacteria, therefore it is important to design and develop novel strategies to tackle this problems. Among various novel strategies, Structurally Nanoengineered Antimicrobial Peptide Polymers (SNAPPs) have been introduced in recent years to overcome this global health care issue and they are found to be more efficient in their performance. Many facile methods are adapted to synthesize complex SNAPPs with required dimensions and unique functionalities. Their unique characteristics and remarkable properties have been exploited for their immense applications in various fields including biomedicine, targeting therapies, gene delivery, bioimaging, and many more. This review article deals with its background, design, synthesis, mechanism of action, and wider applications in various fields of SNAPPs.

**Graphic abstract:**

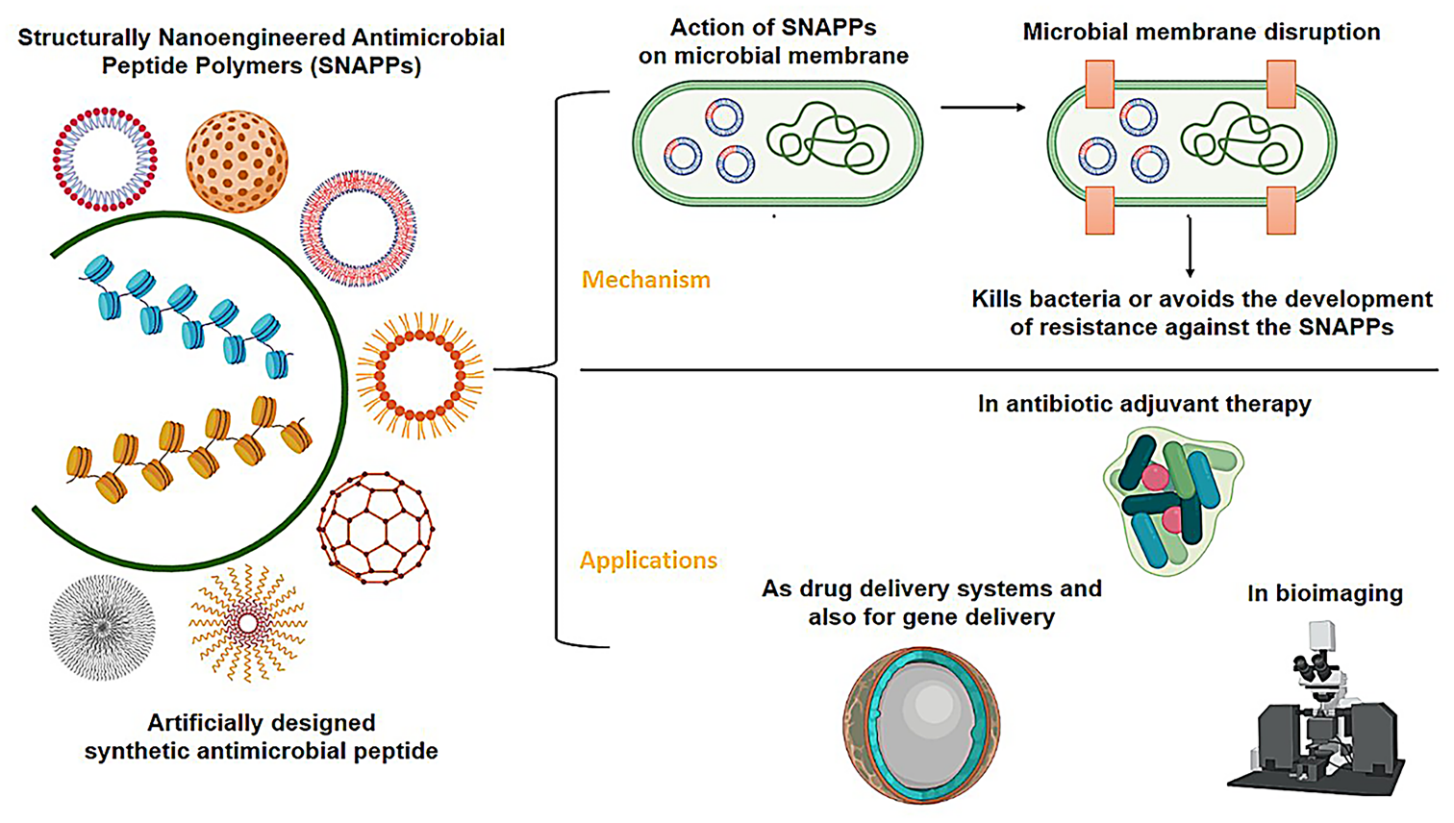

## Introduction

Diseases caused by infectious microbes go on to be the greatest challenge and warning to worldwide health care. Although significant progress has been attained over the past few decades with the introduction of novel and effective antimicrobials, current antimicrobial therapy is still experiencing some major setbacks, including lack of selectivity of usual drugs, unwanted side effects, uneconomical and time-consuming synthetic processes, and more importantly the acquirement of multidrug resistance (Brown and Wright 2016; Namivandi-Zangeneh et al. 2019).

**Fig. 1:**
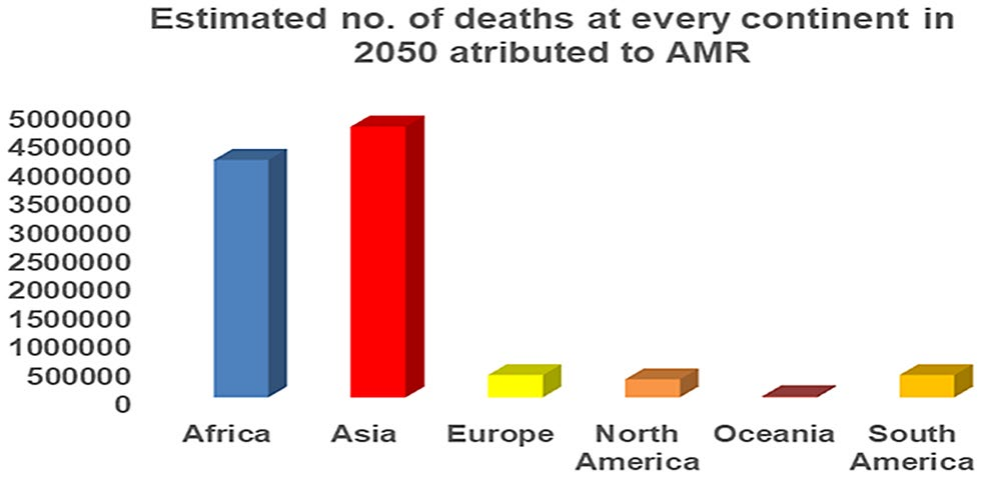
Continent wise estimation of number of deaths by AMR

The term multidrug-resistant (MDR) bacilli cover specifically the pathogens including *Enterococcus faecium, Staphylococcus aureus, Klebsiella pneumoniae, Acinetobacter baumannii, Pseudomonas aeruginosa, and Enterobacter* species which are referred to as ESKAPE pathogens and are being considered as critical and high priority organisms to cause unwanted effects. The success rate posed by conventional antibiotics against these organisms has been proved to be only a handful (Boucher et al. 2009; Mahlapuu et al. 2016; Mukhopadhyay et al. 2020). It has been studied from a recent survey that over 700,000 people are getting affected by drug-resistant microbes each year worldwide. The number is estimated to increase to 10 million in another 50 years if no successful steps are taken (Mahlapuu et al. 2016; Shen et al. 2018). Therefore, it is a necessary prerequisite to design, synthesize and introduce novel antimicrobial agents to overcome or tackle the problems associated with antimicrobial resistance (AMR). Figure 1. depicts a continent-wise estimation of the number of deaths by AMR.

The emergence of drug-resistant microbes and the raising concerns about the usage of antibiotics resulted in the development of antimicrobial peptides (AMPs) which are produced as a first line of defense by many multicellular organisms. AMPs are a class of naturally occuring small peptide molecules and have a wide range of inhibitory actions to directly prevent the growth of organisms like bacteria, fungi, parasites, viruses and even cancer cells. AMPs are classified based on their source, antimicrobial activity, structural characteristics and amino acid composition. Apart from medicine, they also have good application prospects in other fields like food, animal husbandry, agriculture and aquaculture (Mukhopadhyay et al. 2020).

In the past few decades, synthetic replicas of AMPs, a portion of the distinct immune response among all living species have raised focus from researchers as a gifted solution to fight against MDR bacteria (Thomson and Bonomo 2005; Xu et al. 2014; Zu et al. 2014; Gill et al. 2015). These potent, broad spectrum antibiotics are known to exhibit as efficient and novel therapeutic agents which have been proved to be more competitive candidates in antibacterial therapy. There are two types of AMPs based on antimicrobial therapy. The first type refers to inherent antimicrobial polymers which do not involve any modifications while the other type has a need for modifications for the antimicrobial actions. Though having many advantages over common antibiotics, AMPs also faced limited clinical achievements, which may be due to uneconomical manufacturing procedures, inactivation under physiological conditions, a poor profile of pharmacokinetic parameters, and high toxicity in vivo (Ananth et al. 2020; Deslouches et al. 2005; Gill et al. 2015; ). Further, some AMPs exert their actions only under specific experimental conditions and their effect is influenced mainly by forming the interactions between pathogens and tissue-dependent host (Yount et al. 2006; Bechinger and Gorr 2017). These obstructions prevent them from being utilized as efficient systemic therapeutic representatives and many of them are used specifically in topical applications (Rosignoli et al. 2018; Håkansson et al. 2019).

In spite of showing many applications, both natural and synthetic AMPs pose some limitations like damaging the cell membrane of eukaryotes and to cause hemolytic side effects, higher production costs and technical problems, their limited stability at particular pH and so on. In addition, they show decreased activity in the presence of iron and some serum. Further, they are easily hydrolyzed by proteases.

To triumph over the limitations of AMPs, Structurally Nanoengineered Antimicrobial Peptide Polymers (SNAPPs) have been introduced as novel agents having more potency, stability and bioavailability. SNAPPs indicate a new class of synthetic AMP replicas; exist as star-shaped polypeptide nanoparticles containing hydrophilic lysine and hydrophobic valine amino acid residues (Lam et al. 2016a). These are provided with outstanding antimicrobial characteristics and effective even at very low concentrations against many Gram-negative pathogens including multidrug-resistant bacilli to treat the ailments concerned. They are shown to exhibit minimum toxicity and are proved to display superior selectivity on bactericidal action against numerous Gram-negative pathogens compared to conventional antibiotics and linear AMPs (Ng et al. 2013).

In recent years, SNAPPs have been given prime importance in various pharmaceutical fields due to their unique properties and also the degree of functionality that has exposed them as potential candidates to act as successful drug delivery vehicles (Torchilin 2007; Peer et al. 2007; Wu et al. 2015; Chen et al. 2015). Attention has also been focussed on their significance for advanced applications in many fields including emulsification, catalysis, gene delivery, bio-imaging, tissue engineering, and many more (Sulistio et al. 2011; Nakayama 2012). Continued interest in the study of SNAPPs proved them as versatile and unique materials having the potential to employ in high-value pharmaceutical applications. In this article, mainly design, synthesis, mechanism of action, and wider applications of SNAPPs in various fields are discussed.

## Design and synthesis of SNAPPs

SNAPPS in the form of star-shaped peptide polymer nanoparticles, have been recently demonstrated as a new class of antimicrobial agents with superior in vitro and in vivo efficacy and they belong to the class of macromolecular covalently bonded branched architectures (Lam et al. 2016). In their structure, several linear arms are radiating from the central core. They differ from one-dimensional linear polymers in having a higher order of architecture with exclusive properties due to compact 3D structures (Isono et al. 2013). At the core of the structure, there is a multi-functional initiator poly (amidoamine) with primary amines. Lysine and/or valine amino acids are polymerized to the N-terminus of the core to form polymers of varying arm numbers ranging from S16 or S32. There are several types of star structures known, based on composition and sequence, distribution of the arm polymer, the difference in arm species, functional placements, nature, and size of the core structure. There are three prominent approaches available for the synthesis of star shaped SNAPPs which include the core-first, arm-first, and grafting-onto approach. Each of these approaches has merits and demerits of its own.

### Core-first spproach

**Fig. 2:**
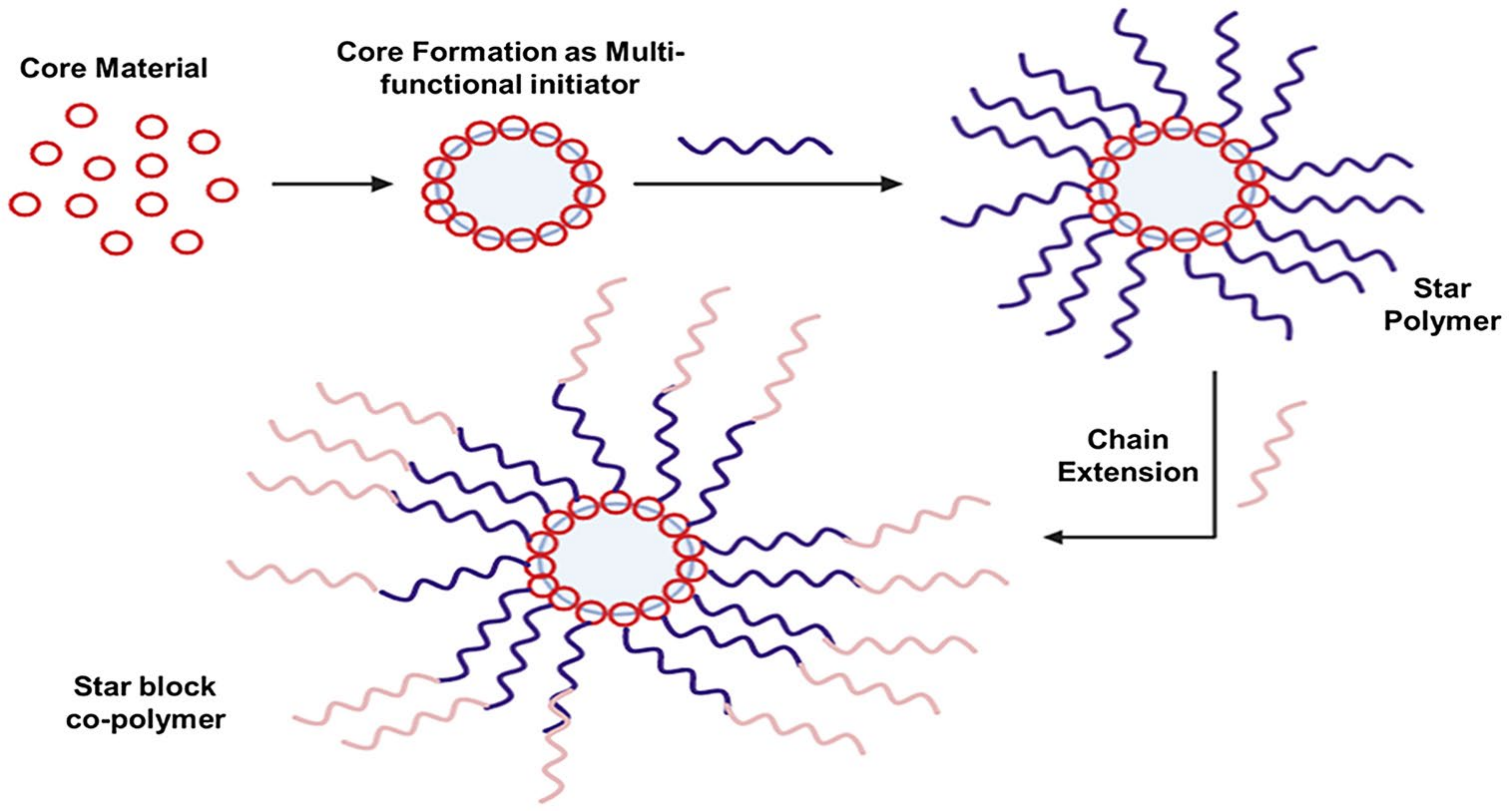
Schematic illustration of the synthetic approaches of SNAPPs via ore-first approach

In this approach, radiating arms are allowed to build from the pre-prepared multifunctional core. The initiating sites on the core must have equal reactivity to obtain polymeric structures with the same arm number and arm length. Further, the rate of the initiation step should be faster than the propagation step. Since there is controlled polymerization, polymers with better control over structure, functionality, and composition can be prepared easily with better yield by involving this approach. The reactions are generally performed under ice-cold conditions to improve the control of polymerization and to avoid side reactions (Vayaboury et al. 2004; Cheng and Deming 2012). But in this approach, sometimes polymers having low arm numbers and a much smaller core domain may be formed which affects both the arm number and core dimension of the resulting star polymer and causes characterization problems. Figure 2. represents the schematic illustration of the synthetic approaches of SNAPPs via the core-first approach.

### Arm-first approach

**Fig. 3:**
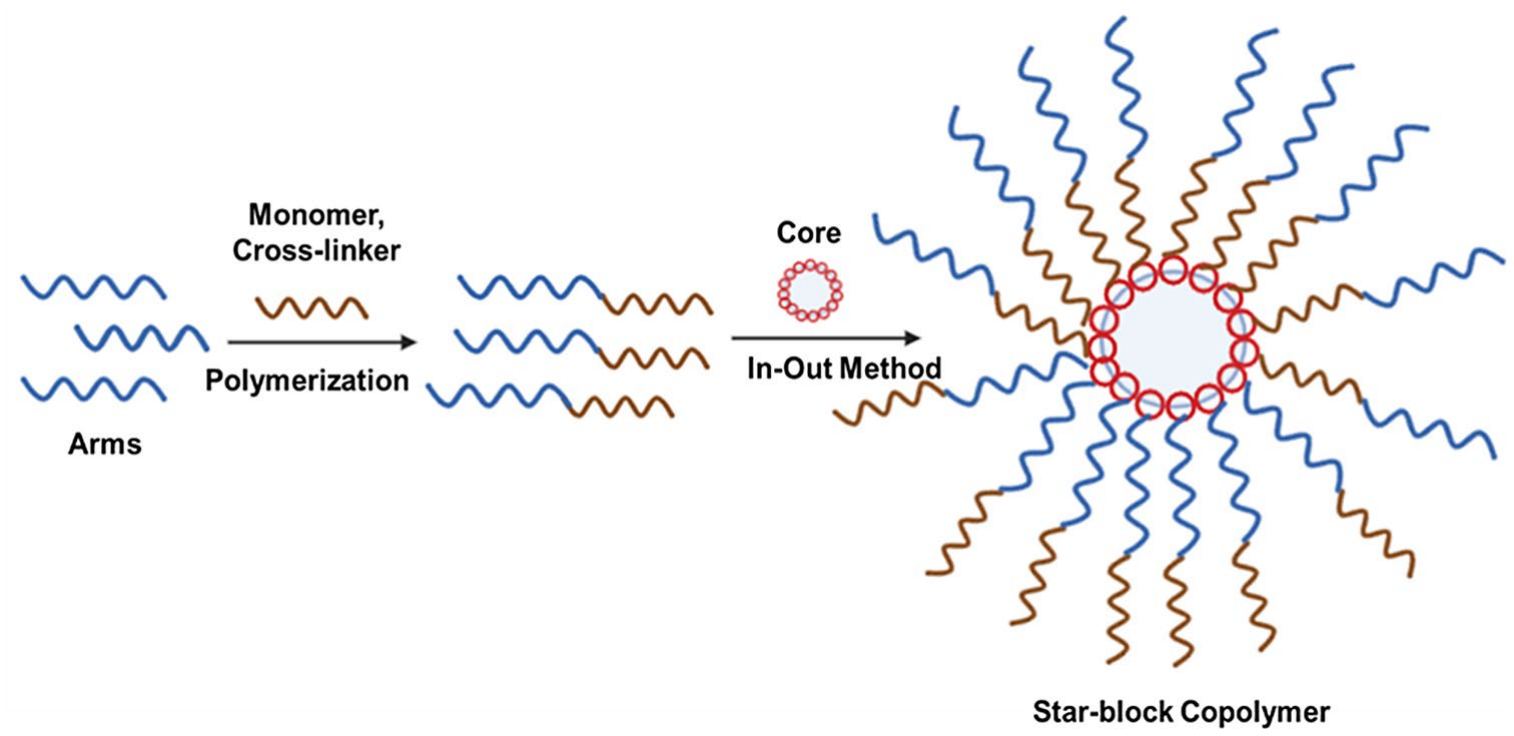
Schematic illustration of the synthetic approaches of SNAPPs via arm first approach

This approach adopts the formation of SNAPPs by crosslinking linear polymers involving coupling reaction in a congregant fashion. The first step is arm formation in which linear polymers act as terminal initiating sites where short cross-linkable block segments are obtained first. This is followed by linking together these linear polymers using coupling polymerization to yield SNAPPs. This approach offers advantages such as facilitating efficient coupling among cross-linkable functionalities, minimal star–star intermolecular cross-linking, better structural control, and generation of polymers with very high molecular weight and large arm numbers (Bapat et al. 2012). Figure 3 shows the schematic illustration of the synthetic approaches of SNAPPs via the arm first approach.

### Grafting-onto approach

**Fig. 4:**
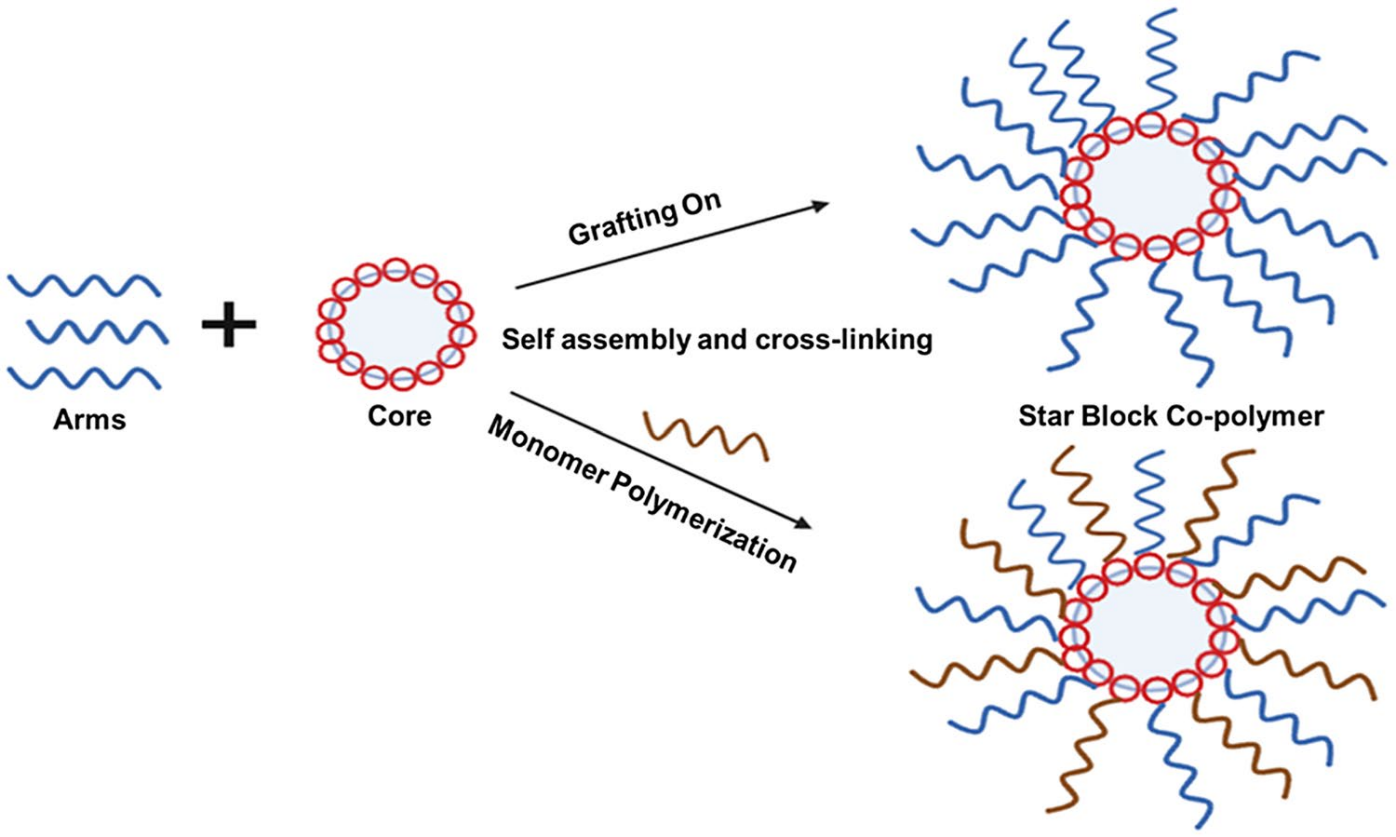
Schematic illustration of the synthetic approaches of SNAPPs via grafting onto approach

In this approach, synthesis and characterization of core and arm can be done independently before SNAPPs formation. Polymers are obtained by coupling reaction of the multifunctional core and arms which act as a balancing reactive terminus. This is followed by post-polymerization end-group modification. The prepared SNAPPs usually have a low arm number and small core size because of the functionality and dimension of the coupling compounds used and also due to steric factors. Figure 4 gives the schematic illustration of the synthetic approaches of SNAPPs via grafting onto the approach.

## Mechanism involved in the SNAPPs formation

The formation of SNAPPs involves various mechanisms such as ring-opening polymerization (ROP), reversible addition − fragmentation chain transfer (RAFT) polymerization, atom transfer radical polymerization (ATRP) and nitroxide-mediated living radical polymerization (NMP).

### Ring-opening polymerization (ROP)

This method involves ROP of cyclic esters and derivatives or N-carboxy anhydride where the later one has been proved to be a more flexible and controls chain-growth polymerization technique for the synthesis of peptide polymers having antimicrobial properties. This method relies on employing amino acids as building blocks to obtain polymers having a close structural resemblance to naturally occurring peptides and proteins, hence specific functionalities can be easily incorporated into polypeptide materials. This approach enables preparing synthetically challenging high molecular weight biopolymers which are better compared to other polymers. Improvements in NCA–ROP have shown a superficial direction for the synthesis of distinct peptide polymers with complex macromolecular architectures, like star polymer nanoparticles though they cannot compete with specific peptide orders attained by solid-phase peptide synthesis (Hawker et al. 2001; Nicolas et al. 2013).

Ring-Opening Metathesis Polymerization (ROMP) is the modified form of the ROP technique that operates by opening the cyclic construction, discharging the ring strain of the monomer, and introducing the monomer into the budding chain. The main advantages of this method include easy flexibility, faster reaction rate, negligible side reactions, and very simple end-group modification (Mota et al. 2013).

### Reversible addition − fragmentation chain transfer (RAFT) polymerization

RAFT polymerization is an extremely flexible method for the synthesis of functional macromolecules with highly complicated topological designs such as multiblock, hyperbranched, gradient, star, and many more. The efficiency of the chain transfer process and the synthesis of RAFT play an important role in influencing the structural integrity of the final polymer like chain-end fidelity and polymer dispersity. Steric congestion acts as a chief feature in the outcome that influences the trials such as addition and fragmentation along with the numerous categories of impurities (Wang et al. 2014; Fischer et al. 2015).

### Atom transfer radical polymerization (ATRP)

ATRP is a method of transition metal-mediated controlled radical polymerization that permits the synthesis of diverse functional polymers with required molecular weights and low disparities (Solomon 2005; Boyer et al. 2011; Moad et al. 2012). The synthesis of brush, comb, SNAPPs, and others have been very well demonstrated and exhibits notable considerations by polymer-researchers with the adaptation of core first, arm-first, or grafting-onto strategies (Sulistio et al. 2011, 2012). A novel group of star polymer shows single-molecule stars made by the intermolecular folding followed by the cross-linking of random copolymers to stabilize “brush-like” structures that act as star arms and hydrophobic moieties that drive the self-folding. But others are for polymerization by post functionalization into vinyl groups to act as a cross-linking point (Liu et al. 2012).

### Nitroxide-mediated living radical polymerization (NMP)

NMP is an old method that has exceptional characteristics like being metal and catalyst-free along with bearable functionalities. Yet, currently, it is not much under use due to its fragile maintenance for the end group and also requires high temperature (> 100 °C) for the reaction to occur (Nakayama 2012; Byrne et al. 2012, 2013; Lam et al. 2016a).

From various studies, the following general observations are drawn from the characterization data of synthesized SNAAPs.

**Fig. 5:**
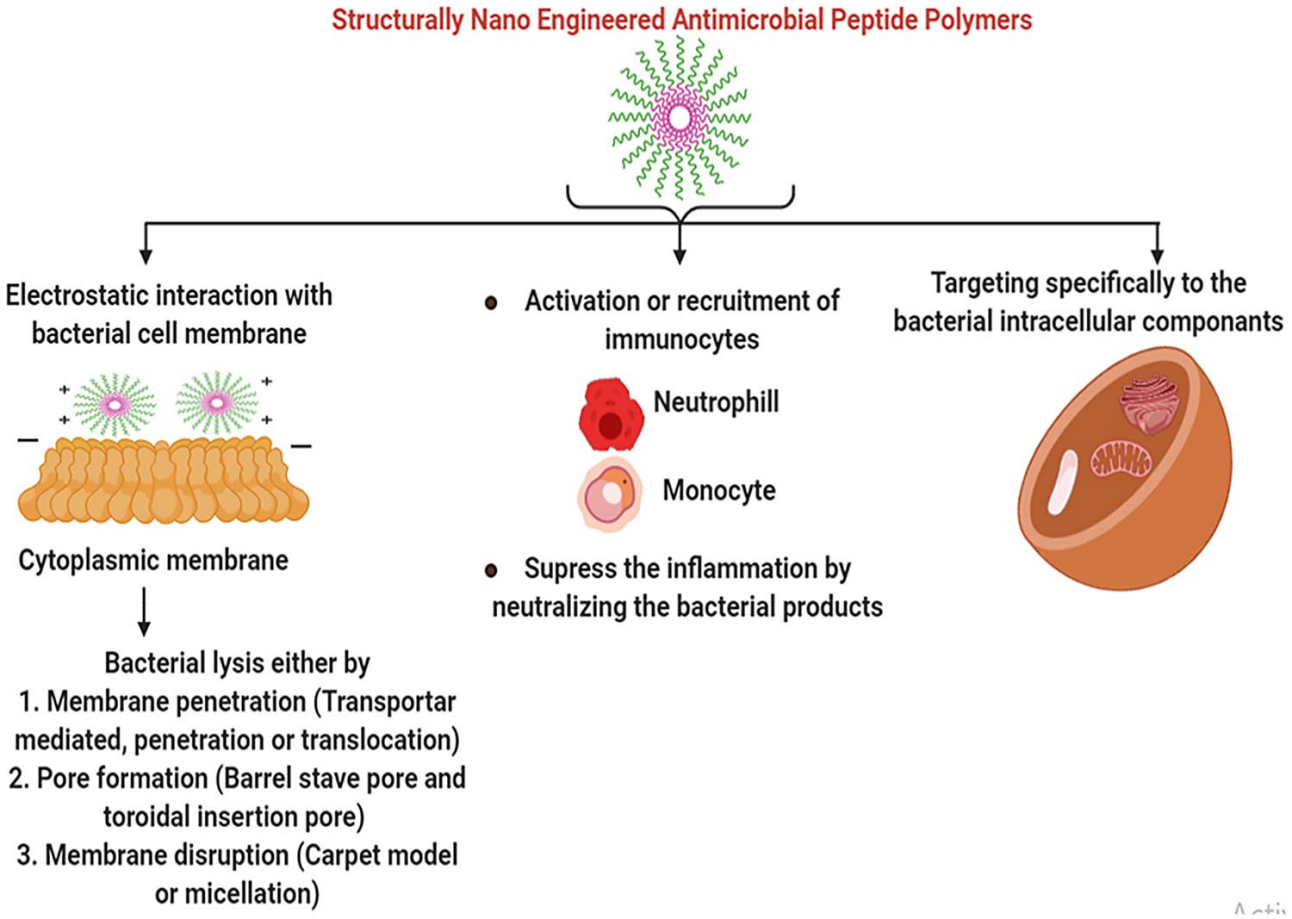
Mechanism of action involved in use of SNAPPs

- Star-shaped SNAPPs with different arm numbers having medium (M) range arm length which was designed in preference to studying the effect of arm number on antimicrobial performance. They exhibited comparable results to the expected values.
- Star-shaped SNAPPs molecular weight is found to be directly proportional to arm number and the hydrodynamic diameter of the stars was also observed to be slightly increasing with arm number.
- Stars with varying arm lengths (S, M, L and VL) have been studied with different degrees of polymerization values. There is an increase in molecular weight and a slight increase in hydrodynamic diameters.
- The surface charge values exhibited similar values obtained across the SNAPPs prepared. Further, any increase in arm number and arm length of the SNAPPs is also found to be increasing with the size of the SNAPPs (Nguyen et al. 2011; Afacan et al. 2012). Figure 5 gives schematic illustration of the arm length of SNAPPs.

## Mechanism of action of SNAPPs

SNAPPs act through a unique, multimodal mechanism involving lipopolysaccharide (LPS) targeting, destabilization /fragmentation of outer membrane, and unregulated ion movements across the membranes in many Gram-negative pathogens, including MDR bacteria. This movement results in membrane disruption along with hyperpolarisation and depolarization of the membrane followed by oxidative stress and production of reactive oxygen species (ROS) (Kohanski et al. 2010). This again is influenced by the arm number and arm length of each SNAPPs. They work literally by tearing cell wall cytoplasmic membrane apart, thus leading to cell death (Lam et al. 2016a).

## Applications of SNAPPs

In recent years, star-shaped nanopolymers and their widespread applications gained more attention among researchers owing to their enhanced stability, biocompatibility, and therapeutic efficiency. They are proved to display a wide range of applications including targeted drug delivery (Sulistio et al. 2011), gene therapy (Lam et al. 2014), bioimaging (Wang et al. 2014), tissue engineering (Mota et al. 2013), and many more. Some of the important and popular applications of SNAPPs are discussed below.

### Antibiotic adjuvant therapy

One of the successful solutions to overcome MDR bacterial infections is to follow synergistic therapy involving co-administration of SNAPPs with other drugs to achieve maximal therapeutic efficacy. This is a promising and economical solution for bacterial infections to surmount the inadequacies of antibiotic immunotherapy and is being extensively used to treat many health disorders including cancer, tuberculosis, and viral infections (Bozkurt-Guzel et al. 2014; Mohamed et al. 2014; Zhang et al. 2014; Namivandi-Zangeneh et al. 2019). Synergistic therapy plays significant roles such as minimizing acquisition of bacterial resistance (Ngu-Schwemlein et al. 2015; De Gier et al. 2016) re-sensitizing MDR bacteria to antibiotics (Goldberg et al. 2013; Amani et al. 2015) and negligible toxic profile (Khalil et al. 2008).

The earlier attempt to combine colistin with another antibiotic to overcome MDR Gram-negative infections was highly unsuccessful due to the emergence of resistant strains. To overcome this problems, SNAPPs were combined synergistically with another drug like ampicillin or gentamicin or silver (Ag+) ions to show counter action against many Gram-negative pathogens. All of them have registered minimum bactericidal concentrations with superior antibacterial efficacies. Further investigation was performed with the synergy of SNAPPs with Doxycycline (Alkawash et al. 1999; Beringer et al. 2012), Imipenem (Rodríguez-Martínez et al. 2009), Tobramycin (De Gier et al. 2016), and so on. All of them were proved to be exhibiting outstanding results with an increase in efficacy compared to when they are administered alone.

SNAPPs have shown great effectiveness against almost all Gram-negative bacteria with the addition of infections that occurred due to CMDR Acinetobacter baumannii. The bacteria did not show any acquisition of resistance to the SNAPPs. SNAPPs showed the death of the bacterial cells by a multimodal mechanism like the destabilization of the outer cell membrane, unregulated movement of ions across the cytoplasmic membrane and apoptotic-like death pathway induction. Its low cost and great effectiveness make it a powerful weapon for combating various MDR bacteria (Lam et al. 2016b).

Star-shaped nanoparticles were prepared by Lam et al. (2016), using SNAPPs and also, bio-nano interaction was studied. Anti-microbial activity of SNAPPs against various Gram-negative pathogen in the different medium was also evaluated to mimic the in vivo conditions. An antagonistic effect was observed in the presence of proteins and salts on the SNAPPs anti-microbial efficacy (Lam et al. 2016c). The relationship between the structure and activity of the SNAPPs was studied for setting the basis for future SNAPPs design and development with improved biological activity. The library of SNAPPs was synthesized by varying the length of arms and their number which can be the prime requirement and investigated for its biocompatibility and the biological antimicrobial activity by performing the antimicrobial assays to examine the mechanism of pathogenic bacteria killing or disruption. An increase in arm length and its number increases its effectiveness, which may be due to the availability of polypeptide arms local concentration with higher alfa-helical content. But meanwhile, it increases the toxicity. Based on calculations of the therapeutic index, it was identified that the polypeptide with 4-arm to 16-arm as best therapeutic moiety. No systemic damage was observed when evaluated for the biocompatibility of SNAPPs with biological activity in mice (Shirbin et al. 2018).

### Drug delivery systems

Due to various problems associated with the delivering pure drug as such, generation of drug carrier which improves the activity of drug by encapsulating it and targeting to the required site. In that, over the past few decades development of well-designed SNAPPs as competent functional encapsulation tools for drug molecules has been a special area of interest (Haag 2004; Torchilin 2007; Peer et al. 2007). SNAPPs have been utilized as the carriers for delivering the guest or drug moieties owing to their unique structural and chemical characteristics. In general, these devices are nonpolar molecules and can be loaded in the hydrophobic core surrounded by a hydrophilic stabilizing crust. SNAPPs with more arm numbers can function as unimolecular drug carriers while using for encapsulation as they do not undergo disintegration into individual polymer under the changes in conditions like pH, ionic strength, and high dilution (Gao 2012) and these are outstandingly stable. Further, the length and block ratio of the arms in these structures can be conveniently regulated. These unique features of SNAPPs make them helpful to achieve effective drug encapsulation and controlled drug release.

Multifunctional integrated systems for encapsulation and drug delivery could be facilitated by introducing different targeting groups into the periphery of SNAPPs where hyperbranched or multifunctional structures provided better results for this purpose (Xiao et al. 2012; Zhao et al. 2013b; Wu et al. 2015). Antineoplastic agents like paclitaxel (Duan et al. 2017), doxorubicin (Schramm et al. 2009) and hydrochlorthiazide (Chen et al. 2011) were successfully encapsulated with superior drug loading content and loading efficiency and there is better cell uptake by tumor tissues. SNAPPs have been proved as effective drug carriers and applicable mainly for sustained, controlled and targeted drug delivery (Wu et al. 2015). This is because of their special characteristics like the solubilizing ability of hydrophobic drugs, high molecular weight, targeting particular physiological sites and easy controlling rate of drug release (Salata 2007). A large number of macromolecules with a high degree of functionality have been prepared and employed as drug delivery vehicles that are capable of reducing the dosage requirements along with minimal undesirable side effects of the drug.

Compared to block copolymer micelles and dendrimers, the use of SNAPPs has registered more efficiency and applications in drug delivery (Wu et al. 2015). These copolymers get self-assembled in aqueous media to form micelle structures in which hydrophobic core domains are enclosed by a hydrophilic periphery. The core domine is capable to solubilize hydrophobic drug molecules while the hydrophilic region is to solubilize the particles in aqueous media. Further, SNAPPs have other advantages as they can also provide an alternative route for drug administration and convenient to administer drugs orally or parenterally. Demonstration on encapsulation of doxorubicin in SNAPPs was performed in which the drug was made to conjugate with the aldehyde groups in the star core via an imine linkage and resulting in controlled release of the drug at lower pH (Nasr et al. 2015). It was shown that the SNAPPs could accumulate better in the tumour cell with longer plasma circulation time and confirmed that these polymers potential enough for improved delivery of therapeutic agents in vivo. Drugs such as paclitaxel (Duan et al. 2017), progesterone (Jones et al. 2003), furosemide (Schramm et al. 2009), hydrochlorthiazide (Chen et al. 2011), etoposide (Wang et al. 2005) and 5-fluorouracil (Aryal et al. 2009) have also been successfully encapsulated in the core of SNAPPs to explore their releasing efficiencies.

Moreover, this type of delivery system must be stable during systemic circulation in blood to minimise the leakage of the encapsulated drug before reaching the target tissue and this depends again on the local environment (Chen et al. 2008; Cabral and Kataoka 2010). Later, active targeting by functionalizing drug carriers with antibodies, peptides and small molecules have been adapted to improve efficiency which can distinguish exact receptors in pathological tissues and there is a severe reduction in adverse effects observed (Prabaharan et al. 2009; Yang et al. 2010).

### For gene delivery

Various genetic disorders like cancer, diabetes, blindness, cystic fibrosis and parkinson’s disease can be treated successfully with the application of gene therapy (Grigsby and Leong 2010). Therapy or treatment provided by this mode is dependent on the delivery of genetic materials such as plasmid DNA, siRNA and microRNA into cells, as these negatively charged hydrophilic materials cannot efficiently pass through hydrophobic membranes which are also negatively charged. Further, these materials can undergo enzymatic degradations before reaching the nucleus; hence it is necessary to encapsulate them (Hancock and Sahl 2006; Zhou et al. 2010). The earlier attempts to use viral carrier vectors for encapsulation were unsuccessful clinically due to various reasons like possible immune responses, the amount and size of genetic materials and high production cost. Hence, the cationic SNAPPs have been employed as transfection vectors to complex electrostatically with the negatively charged nucleic acids. The aqueous solubility and biocompatibility were improved with polyethylene glycol (PEG) as a common chemical group connected to polymeric vectors (Cloninger 2002) which depends upon molecular weight, architecture, degree of branching, and charge density of SNAPPs.

An extensive study on gene therapy in relation to SNAPPs based on peptide-functionalized polymers or polypeptides has been carried out by many research teams (Fichter et al. 2008; Zhao et al. 2013a) and the results were documented in terms of size and surface charges that the star-shaped polymers can complex efficiently compared to the linear ones. It was also showed that the size of polymers plays a major role in systemic and intratumoral distributions with their size range of 10 − 30 nm exhibiting deep tissue penetration (Lee et al. 2010; Tang et al. 2012) and decreased blood clearance (Perrault et al. 2009).

Newer type core-shell SNAPPs have also been synthesized which can complex with many nucleic acid molecules. The chain extension with PEG on star polymer having N-(2-aminoethyl) methacrylamide hydrochloride (AEMA.HCl) cationic core has displayed better colloidal stability, neutral zeta potentials and minor cellular toxicity which provides a modified carrier for efficient siRNA delivery. SNAPPs that can respond to stimuli like pH (Kim et al. 2009; Guo et al. 2010), redox potential (Kamada et al. 2010; Terashima et al. 2014, 2015), light (Nyström et al. 2011; Boyer et al. 2012), temperature (Tan et al. 2014; McKenzie et al. 2016) and enzymatic changes (Byrne et al. 2012; Thornton et al. 2013) have been recently developed and one of the popular area for targeted drug and gene delivery applications.

### Interfacial stabilizing agents

Recent studies suggest that SNAPPs having many arms and a crowdedly cross-linked core are established as efficient interfacial stabilizing agents for emulsion systems (Qiu et al. 2011; Li et al. 2012). The flexible polymeric chains spread out from the central core can be readily deformed and can act as either pickering stabilizers (particle-like) or established asymmetric surfactants (molecule-like) (Binks and Lumsdon 2001). When demixing of the arm occurs from solubility differences, traditional asymmetric type structures are formed in the form of much larger sized spheres having characteristics of surfactant. Hence, SNAPPs can be stimuli-responsive which can distinguish a stable emulsion from an unstable state. Reversible emulsification demulsification processes on stimuli-responsive core cross-linked star (CCS) polymers have been studied and shown that the thermoresponsive nature of these polymers is accountable for thermally triggered demulsification of polymer-stabilized emulsions (Binks and Lumsdon 2001). This sort of control and adjustability over temperature for destabilization can be conveniently applicable in environments like targeted drug delivery.

The conversion of W/O system into O/W type and vice versa depends mainly upon pH. At lower pH, the arms are more protonated, become cationic, and prefer their higher solubility in water and lesser in an oil phase. Hence the formation of O/W emulsion is favoured where the polymers go mainly into the aqueous continuous phase (Golemanov et al. 2006). With an increase in pH, there is a preferential formation of W/O type emulsion. It was also demonstrated that stabilization of emulsions can be possible at a very low concentration of polymers and in particular, SNAPPs which are larger with more radiating arms could stabilize emulsions at lower concentrations efficiently. These parameters are highly helpful to overcome the formulation problems in the study of emulsions.

### Bioimaging

By following the biodistribution and targeting efficiency of a drug delivery system, an in vivo studies have been performed to know the cell migration and nature of the disease. An imaging with clinical diagnostic methods/agents help in assessing the state and extent of the disease before deciding for initial therapy (Adkins et al. 2012). The distinctive properties and structural characteristics of SNAPPs make them appropriate as fluorescent explorers, contrast agents and in vitro diagnostic systems (Fukukawa et al. 2008; Wang et al. 2012; Bagby et al. 2012).

To determine the effect of SNAPPs on Gadolinium Magnetic Resonance Imaging (Gd^3+^MRI) contrast agents, macromolecular ligands were chelated to complex Gd^3+^ and the results were compared by studying reflexivity properties (Li et al. 2012). The star nano gel exhibited a relaxation rate much higher than in the commercial Gd^3+^ MRI contrast product. Poly acrylate-based SNAPPs with bimodal imaging agents which is the combination of fluorescent and magnetic resonance features have also been developed (Adkins et al. 2012). Later, SNAPPs were structurally altered with dopamine analogs and made into chelate formation with lanthanides like Gd^3+^ and Eu^3+^. High molecular weight polymer drug carriers with branched star-like structural design have been proved to exhibit higher accumulation in tumor tissues due to better permeability and retention effect (Ulbrich et al. 2003; Etrych et al. 2008).

## Summary and future prospectives

Currently, SNAPPs, in the form of star-shaped polymers present an emerging novel approach for the treatment of various infections associated with antimicrobial resistance. The unique properties of SNAPPs make them more successful and widely acceptable for the various applications of the pharmaceutical field and also help in delivering the drug molecules with minimal toxicity and greater therapeutic effect. In the current review, we have covered the detailed synthesis and mechanism behavior of the SNAPPs. Also, various novel applications of SNAPPs are included by discussing the case studies.

In the future, with the help of various approaches, the effective, less toxic and nanomaterial sized SNAPPs can be given priority to provide permanent solutions for microbial resistance developed infections. Though having many advantages over other polymers and formulations, the commercial utility of these precious SNAPPs is awarded less attention. More focus can be provided for their commercial value from researchers by keeping parallel coordination with those from other disciplines and industries. It is highly necessary to continue extensive research in the development of efficient star-polymers to acquire highly economical and still better reproducible outcomes. Further modulations of functionalities in SNAPPs development and their role in therapeutic value with minimal impurities and utility in many other important fields can be kept as a futuristic goal for the forthcoming research activities.
